# Molecular and morphological evidence reveals a new genus of the subfamily Heteropterinae (Lepidoptera, Hesperiidae) from China

**DOI:** 10.3897/zookeys.1055.68640

**Published:** 2021-08-05

**Authors:** Yongxiang Hou, Hideyuki Chiba, Lijuan Zhu, Zhou Chang, Lijun Ma, Siyao Huang, Min Wang, Xiaoling Fan

**Affiliations:** 1 Department of Entomology, College of Plant Protection, South China Agricultural University, Guangzhou, Guangdong 510642, China South China Agricultural University Guangzhou China; 2 B. P. Bishop Museum, 1525 Bernice Street, Honolulu, Hawaii, 96817-0916, USA B. P. Bishop Museum Honolulu United States of America; 3 State Key Laboratory of Genetic Resources and Evolution, Kunming Institute of Zoology, Chinese Academy of Sciences, Kunming, Yunnan 650201, China Kunming Institute of Zoology, Chinese Academy of Sciences Kunming China; 4 Institute of Plant and Environmental Protection, Beijing Academy of Agriculture and Forestry Sciences, Beijing 100097, China Beijing Academy of Agriculture and Forestry Sciences Beijing China

**Keywords:** *
Carterocephalus
*, *
Pulchroptera
*, new combination

## Abstract

Molecular phylogenetic analysis indicates that the genus *Carterocephalus* is not monophyletic. Based on combined molecular and morphological evidence, we propose a new genus, *Pulchroptera* Hou, Fan & Chiba, **gen. nov.**, for *Pamphilapulchra* Leech, 1891. The adult, wing venation, and male genitalia of *Pulchropterapulchra***comb. nov.**, *Carterocephaluspalaemon*, and related genera are illustrated.

## Introduction

In recent years, the molecular phylogeny of the family Hesperiidae has attracted the attention of an increasing number of researchers (Warren et al. 2008, 2009; Sahoo et al. 2016; Toussaint et al. 2018; Cong et al. 2019; Li et al. 2019; Liu et al. 2020). At the subfamily level, however, the phylogeny of the family Hesperiidae has yet to be established, and multiple new subfamilies (Zhang et al. 2019, 2020) and genera (Fan et al. 2016; Huang et al. 2016, 2019; Cong et al. 2019; Li et al. 2019) have been proposed in recent years.

Although Heteropterinae, including 13 genera from Africa, was established already by Aurivillius (1925), subsequent authors did not recognise the subfamily, presumably because it was a mixture of genera and species assigned to the subfamilies Hesperiinae as well as Heteropterinae in the current taxonomy. Evans, somehow, proposed three different genus group names for taxa of these skippers, namely the *Astictopterus* group for the African taxa (Evans 1937), the *Heteropterus* group for the European and Asian taxa (Evans 1949), and the *Carterocephalus* group for the American taxa (Evans 1955), all of which he considered a part of the subfamily Hesperiinae. This arrangement was accepted in subsequent taxonomic works until Higgins (1975) and Scott and Wright (1990) restored the subfamily Heteropterinae. Recent molecular studies strongly support the monophyly of the subfamily (Warren et al. 2008; Sahoo et al. 2016, 2017; Toussaint et al. 2018, 2020; Cong et al. 2019; Li et al. 2019; Zhang et al. 2019; Liu et al. 2020). Currently, Heteropterinae includes the following 13 genera: *Heteropterus* Dumeril, 1806; *Carterocephalus* Lederer, 1852; *Butleria* Kirby, 1871; *Argopteron*, 1893; *Dalla* Mabille, 1904; *Leptalina* Mabille, 1904; *Metisella* Hemming, 1934; *Dardarina* Evans, 1937; *Hovala* Evans, 1937; *Piruna* Evans, 1955; *Freemaniana* Warren, 2001; *Ladda* Grishin, 2019; and *Willema* Grishin, 2019. Moreover, the most recent studies indicate that all these genera are monophyletic (Cong et al. 2019; Toussaint et al. 2020).

The genus *Carterocephalus* includes more than 20 species distributed in the Holarctic and Oriental regions. However, a cursory inspection of the male genitalia indicates that *C.pulchra* (Leech, 1891) is not a congener of the type species *Papiliopalaemon* Pallas, 1771. Indeed, the findings of our morphological and molecular phylogenetic studies have revealed closer relationships with species in the genera *Heteropterus* and *Leptalina*. Accordingly, we consider that *Carterocephaluspulchra* should be placed in a new genus.

In the present study, we sought to assess the monophyly of the genus *Carterocephalus* and its relationship with other genera of Heteropterinae. On the basis of the evidence obtained, we describe a new genus.

## Materials and methods

### Morphological examination

For the morphological study, we followed the methods described by Fan et al. (2010). To examine wing venation, wings were removed from the thorax and cleaned with a 1:1 mixture of bleaching liquid (Blue Moon, Guangzhou, China) and water for approximately 3 to 4 min. Photographs of the wing venation and male genitalia were taken using a Keyence VHX-5000 digital microscope (Keyence, Osaka, Japan).

### Taxon sampling

We sampled specimens from all genera listed in the subfamily Heteropterinae (Warren et al. 2008, 2009; Cong et al. 2019; Toussaint et al. 2020), including as many species as possible. We used a total of 44 specimens of 38 species in 13 genera as ingroup taxa, along with 12 species from other subfamilies (Coeliadinae, Pyrginae, Eudaminae, Euschemoninae, Barcinae, Trapezitinae, and Hesperiinae) as outgroup taxa. Among these specimens, 31 were newly sequenced in this study, with the remaining sequences being obtained from the GenBank database along with supplementary data presented by Sahoo et al. (2016) and Toussaint et al. (2020). The respective voucher specimens and additional information are listed in Suppl. material 1: Table S1. Vouchers bearing codes beginning with the abbreviation SCAU have been deposited in the collection of South China Agricultural University (**SCAU**), Guangzhou, China, and the specimens (JU19), (Dalla), and (SZSMETI) are retained in the private collections of J. Uehara, H. Chiba, and S. Sáfián, respectively.

### Laboratory protocols

DNA was extracted from two or three legs of dried adult specimens using a TIANamp Genomic DNA Kit (Tiangen, Guangzhou, China) following the manufacturer’s instructions. We amplified a single mitochondrial gene (658 bp of COI) and three nuclear genes (1066 bp of EF-1α, 610 bp of RPS5, and 403 bp of Wingless), for a total of 2737 bp. The primers used to amplify each gene were synthesised by Sangon Biotech (Shanghai, China) and are shown in Suppl. material 2: Table S2. DNA amplification was performed in 20-µL reaction volumes containing 1 µL of template DNA, 0.8 µL of each primer (10 µM), 10 µL of 2× EasyTaq PCR superMix (+dye) (Transgen, Beijing, China), and 7.4 µL of ddH_2_O. The amplification protocol adopted is the one described by Huang et al. (2019). Sequencing of the amplicons thus obtained was performed by Sangon Biotech (Shanghai, China) and Tsingke Biological Technology (Beijing, China), and new sequences have been deposited in GenBank (Suppl. material 1: Table S1).

### Phylogenetic analyses

Sequences were aligned using Clustal W (Thompson et al. 1997) and edited manually using MEGA 7.0 (Kumar et al. 2016). Gene data from Cong et al. (2019) were extracted from the genomic assembly in IDBA-UD (Peng et al. 2012). PartitionFinder v2.1.1 (Lanfear et al. 2012, 2016; Guindon et al. 2010) was used to select the optimal codon partitioning scheme under Akaike information criterion correction (AICc) (Suppl. material 3: Table S3). We inferred the phylogenetic trees using two methods, namely maximum likelihood (ML) and Bayesian inference (BI), for which we used the partition scheme produced by PartitionFinder. ML analyses were performed using IQ-TREE (Nguyen et al. 2015) as implemented in the IQ-TREE web online server (iqtree.cibiv.univie.ac.at, Trifinopoulos et al. 2016), with branch support values evaluated based on 1000 replicates for ultrafast bootstrap (UFBoot) (Minh et al. 2013) and SH-aLRT (Guindon et al. 2010). BI analyses were performed using the CIPRES Science Gateway (https://www.phylo.org/) (Miller et al. 2010) with Markov Chain Monte Carlo (MCMC) randomisation in MrBayes using XSEDE 3.2.6 (Ronquist et al. 2012). Reversible-jump MCMC was used to facilitate sampling across the entire subduction rate model. We conducted two independent MCMC runs, with four Markov chains (5 × 10^6^ generations) for each analysis, of which the initial 25% of samples were discarded as burn-in. Bayesian posterior probabilities (PP) were used to evaluate branch support, and trees were visualised using FigTree v1.4.0.

## Results and discussion

### Phylogenetic relationships

**Figure 1.**
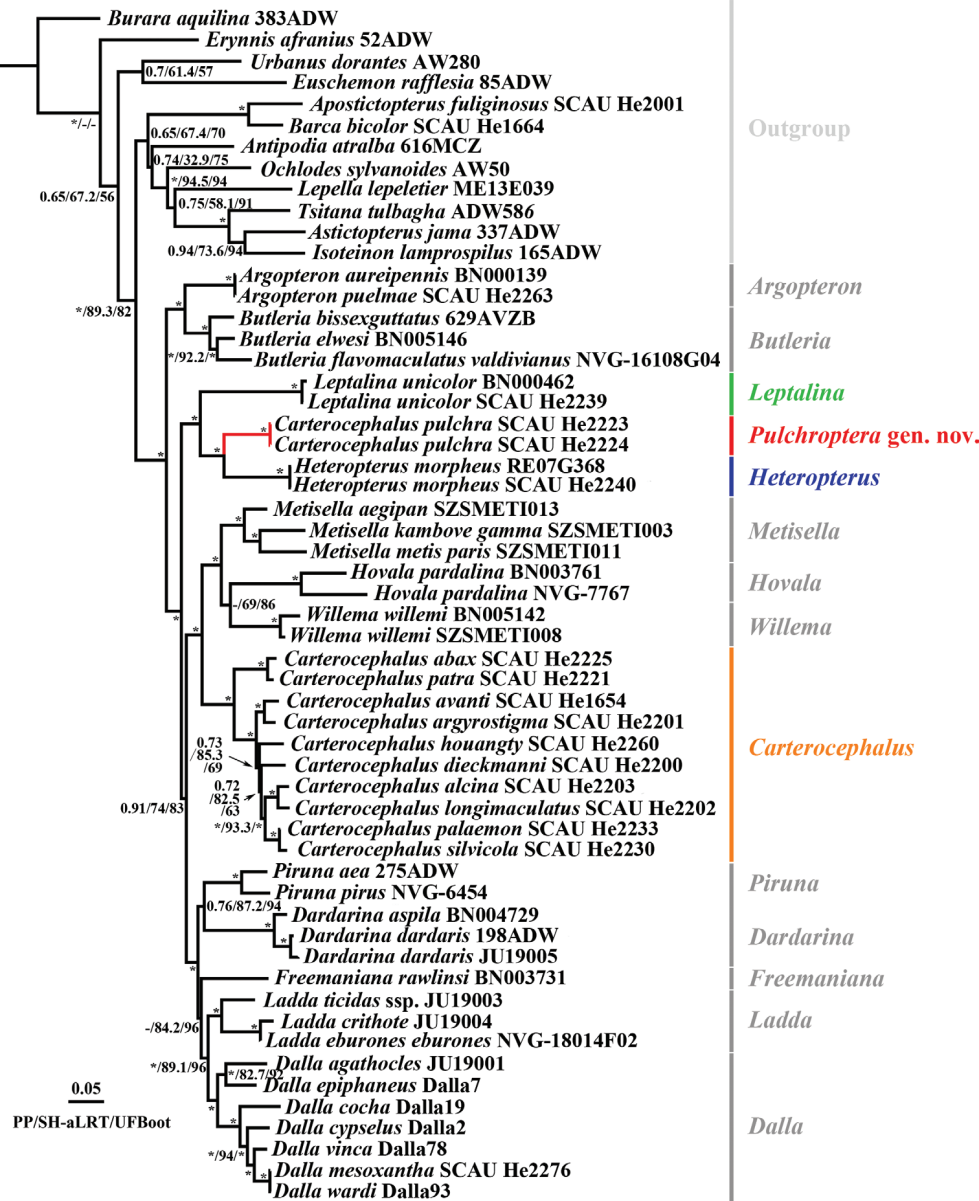
Maximum likelihood phylogenetic tree of the subfamily Heteropterinae. Values at nodes represent the posterior probabilities (PP) of BI analyses values, SH-aLRT values (SH-aLRT), and Ultrafast bootstrap support values (UFBoot) of the maximum likelihood analysis. * indicates that one of the values at a node exceeds the standard (PP ≥ 0.98, SH-aLRT ≥ 95, UFBoot ≥ 98). When the three node values all reach the standard, only one * is displayed. – indicates that the node was not recovered in the ML or BI tree.

The topological structures of the concatenated dataset inferred by ML and BI analyses were found to be generally consistent and strongly supported at most nodes (PP ≥ 0.98, SH-aLRT ≥ 95, UFBoot ≥ 98) (Fig. 1). Moreover, the two analyses provided strong support for the monophyly of Heteropterinae (PP = 1, SH-aLRT = 99.9, UFBoot = 100), which excludes the genera *Apostictopterus*, *Barca*, *Lepella*, and *Tsitana* originally assigned to this subfamily, and is consistent with the findings of the most recent studies (Toussaint et al. 2018, 2020; Cong et al. 2019; Zhang et al. 2019). Within the subfamily Heteropterinae, four major clades were differentiated, with 14 well-supported monophyletic subclades, corresponding to the 13 currently recognised genera and the *Carterocephaluspulchra* clade. Certain results were consistent with those of previous studies (Cong et al. 2019; Toussaint et al. 2020): (1) of the 13 genera, 12 genera, excluding *Carterocephalus*, were monophyletic; (2) *Argopteron* and *Butleria* formed a strongly supported monophyletic group (PP = 1, SH-aLRT = 99.1, UFBoot = 100) that is sister to all other genera in Heteropterinae (PP = 1, SH-aLRT = 98.3, UFBoot = 99); (3) *Carterocephalus*, excluding the species *C.pulchra*, was sister to the clade containing *Metisella*, *Hovala*, and *Willema* with strong support (PP = 1, SH-aLRT = 99.5, UFBoot = 100); and (4) *Piruna*, *Dardarina*, *Freemaniana*, *Ladda* and *Dalla* formed a strongly supported monophyletic clade (PP = 1, SH-aLRT = 98.8, UFBoot = 99). Two findings, however, are inconsistent with those reported previously. Firstly, *Piruna* is sister to *Dardarina* (PP = 0.76, SH-aLRT = 87.2, UFBoot = 94), as opposed to sister to the four genera *Dardarina*, *Freemaniana*, *Ladda*, and *Dalla*. Based on the morphology of the male genitalia (Evans, 1955), *Piruna* shows a relatively close similarity to *Dardarina*, whereas species of *Dalla* show extensive variation. However, previous molecular phylogenetic studies, as well as our own, sampled only some representatives of *Dalla*. Accordingly, the monophyly of *Dalla* as well as the relationships among these five genera should be subjected to further studies. Secondly, we found that *Carterocephalus* is not a monophyletic group, given that the 11 species analysed in the present study were recovered in two distinct clades, with *C.pulchra* clustering with *Leptalina* and *Heteropterus* with strong support (PP = 1, SH-aLRT = 97.7, UFBoot = 100). The other ten species, including the type species *C.palaemon*, were recovered as a strongly supported monophyletic clade.

Although in this study we focused on relationships among the genera of Heteropterinae, it is worth mentioning that certain intra-generic relationships, namely, those between *C.abax* Oberthür, 1886 and *C.patra* Evans, 1939, *C.avanti* (de Nicéville, 1886) and *C.argyrostigma* (Eversmann, 1851), *C.longimaculatus* Hou, Fan & Chiba, 2021 and *C.alcina* Evans, 1939, *C.palaemon* (Pallas, 1771) and *C.silvicola* (Meigen, 1828) are strongly supported. As described by Toussaint et al. (2020), despite the lack of strong support (PP = 0.73, SH-aLRT = 85.3, UFBoot = 69), *C.houangty* and *C.dieckmanni* were clustered in a clade comprising *C.palaemon*, *C.silvicola*, *C.longimaculatus*, and *C.alcina*. In our previous study (Hou et al. 2021), we established that *C.dieckmanni* is sister to *C.abax* and *C.patra*. However, owing to an oversight, the names *C.dieckmanni* and *C.argyrostigma* were confused, which explains the discrepancy compared with the results reported herein. Accordingly, to determine relationships more comprehensively in the genus *Carterocephalus*, we ideally need to undertake additional and more extensive sampling.

**Figure 2.**
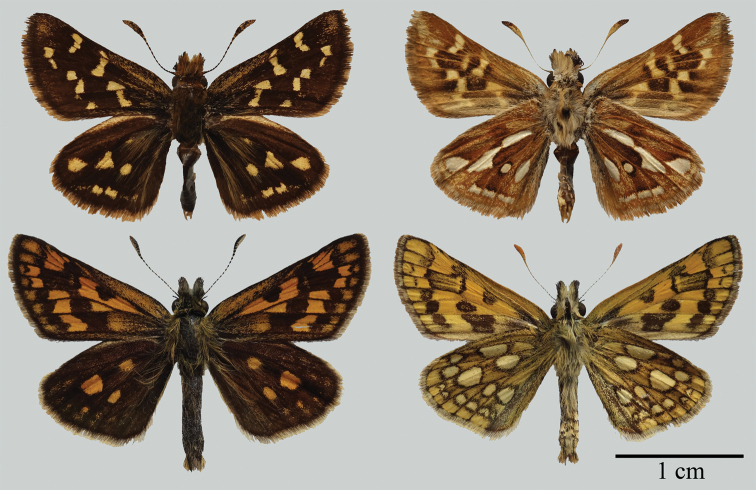
Male adults of the two skippers. Above: *Pulchropterapulchra* (Leech, 1891) comb. nov. from Kunming, Yunnan, China; below: *Carterocephaluspalaemon* (Pallas, 1771) from Moscow, Russia.

**Figure 3.**
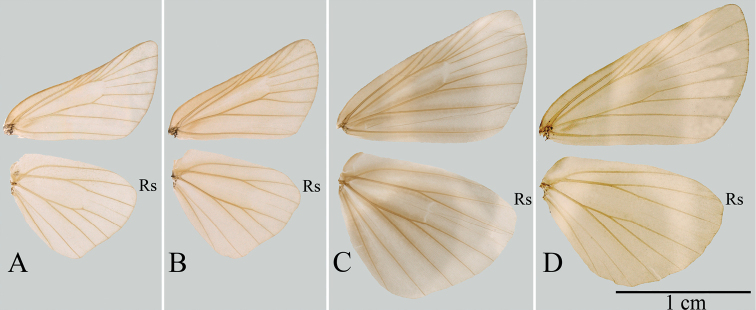
Wing venation of four genera of Heteropterinae**A***Pulchropterapulchra* (Leech, 1891) comb. nov. **B***Carterocephaluspalaemon* (Pallas, 1771) **C***Heteropterusmorpheus* (Pallas, 1771) **D***Leptalinaunicolor* (Bremer & Grey, 1852).

**Figure 4.**
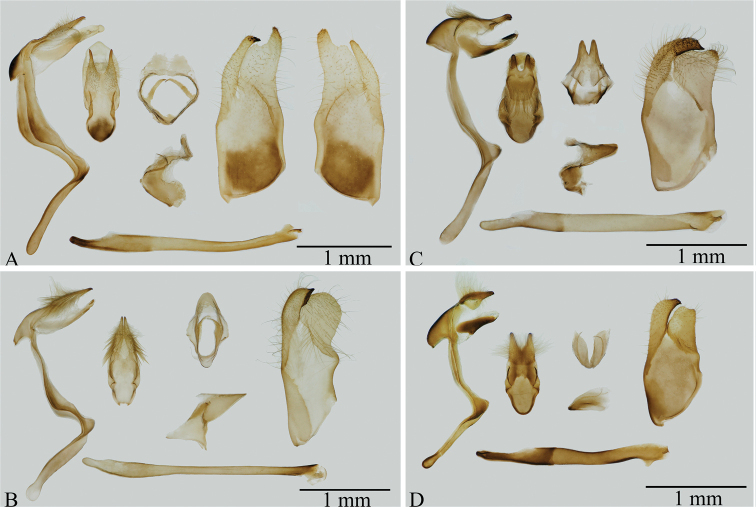
Male genitalia of four genera of Heteropterinae**A***Pulchropterapulchra* (Leech, 1891) comb. nov. **B***Carterocephaluspalaemon* (Pallas, 1771) **C***Heteropterusmorpheus* (Pallas, 1771) **D***Leptalinaunicolor* (Bremer & Grey, 1852).

Morphologically, although *C.pulchra* is similar to the type species of *Carterocephalus* with respect to wing shape and pattern (Fig. 2), the origin of vein R_s_ on the hindwing is located nearly midway between the termen and the base in *C.pulchra*, *Heteropterus*, and *Leptalina*, whereas in other species of *Carterocephalus* the origin of vein R_s_ is closer to the termen than to the base (Fig. 3). With regards to the male genitalia, the uncus in *C.pulchra*, *Heteropterus*, and *Leptalina* is deeply bifurcated, with arms distant from each other, whereas in the type species of *Carterocephalus* the uncus bifurcates with arms closely aligned (Fig. 4). These morphological similarities would accordingly appear to indicate that *C.pulchra* is more closely related to *Heteropterus* and *Leptalina* than to other species of *Carterocephalus*. Of these related genera, *C.pulchra* is autapomorphous with respect to its male genitalia. Notably, the gnathos is weakly sclerotized, membranous, and rounded at the tip, the valvae are asymmetrical, and the juxta is a heart-shaped ring with a narrow and long latero-central process. In summary, we propose a new genus, *Pulchroptera* Hou, Fan & Chiba gen. nov., for the *Carterocephaluspulchra* clade based on its autapomorphies and molecular evidence.

#### Pulchroptera

Taxon classificationAnimaliaLepidopteraHesperiidae

Hou, Fan & Chiba gen. nov.

D69196C8-120B-5455-B39A-41835A35E412

http://zoobank.org/3C184FA2-423E-43F5-B77A-5D06C5639245

Figures 2 , 3 , 4

##### Type species.

*Pamphilapulchra* Leech, 1891

##### Description.

Forewing length 11–12 mm. Antennae approximately half the length of forewing; nudum 8 on apiculus, dark brown. Palpi on second segment long and erect, yellow with long black hairs; on third segment black, thick, short, and porrect. Wing venation (Fig. 3): forewing: length of discoidal cell almost equal to 2/3 forewing length, Sc ends at 1/2 forewing length; origin of vein R_4_ before vein R_5_; origin of vein M_2_ in middle of veins M_1_ and M_3_; veins CuA_1_, CuA_2_, and 1A+2A almost parallel to each other; origin of vein CuA_2_ nearly midway between vein CuA_1_ and base. Hindwing: costa longer than dorsum; length of discoidal cell almost equal to 3/5 hindwing; origin of vein Rs midway between base and termen; origin of vein M_2_ slightly nearer M_1_ than M_3_. Wing ground colour and wing patterns: upper side dark brown with small yellow spots in central and submarginal areas; underside light brown, forewing patterns similar to upper side, hindwing with small silvery spots in spaces Rs, M_3_, CuA_1_, and CuA_2_, and with a silvery longitudinal central streak. Mid and hind tibiae each with pair of spurs. Male genitalia: Tegument small and narrow, constricted at middle in dorsal view; uncus deeply bifurcated, V-shaped dorsally; gnathos long and wide, longer than tegument, membranous, undivided from basal 1/3; saccus long; valvae asymmetrical, bifid, distal end of left valva more sclerotized than right valva; aedeagus long, subzonal sheath shorter than suprazonal sheath, ratio of subzonal sheath to suprazonal sheath approximately 1:2, vesica with cornuti; juxta a heart-shaped ring with membranous extensions dorsally.

##### Remarks.

The new genus superficially resembles *Carterocephalus* Lederer, 1852, although it is distinguishable from the latter with regards to the following characters: hindwing undersides with silver spots, a deeply bifurcated V-shaped uncus, juxta a heart-shaped ring, and valvae asymmetrical.

The new genus contains only the type species *Pulchropterapulchra* (Leech, 1891) comb. nov., with the nominotypical subspecies and a further subspecies, *Pulchropterapulchraops* (Grum-Grshimaïlo, 1891) comb. nov. According to the description of Evans (1949), in *Pulchropterapulchrapulchra* comb. nov. the upper side of the hindwing has a cell spot and the submarginal markings are notably more conspicuous, whereas in *Pulchropterapulchraops* comb. nov. the upper side of the hindwing lacks a cell spot and has conspicuous submarginal markings. Whether the subspecies status of the latter is valid is subject to further verification.

##### Etymology.

The name of the genus is taken from the specific epithet of the type species ‘pulchr-’, meaning beautiful, and ‘optera’, meaning wing. The gender is feminine.

##### Distribution.

*Pulchropterapulchrapulchra* comb. nov.: China (Sichuan, Yunnan)

*Pulchropterapulchraops* comb. nov.: China (Gansu, Qinghai, Xizang)

## Supplementary Material

XML Treatment for Pulchroptera

## Supplementary materials

Supplementary material 1 Table S1 Data type table Explanation note Label information and GenBank accession numbers of the specimens used in this study. https://binary.pensoft.net/file/574783This dataset is made available under the Open Database License (http://opendatacommons.org/licenses/odbl/1.0/). The Open Database License (ODbL) is a license agreement intended to allow users to freely share, modify, and use this Dataset while maintaining this same freedom for others, provided that the original source and author(s) are credited.Author: Yongxiang Hou

Supplementary material 2 Table S2 Data type table Explanation note Primers used in this study. https://binary.pensoft.net/file/574784This dataset is made available under the Open Database License (http://opendatacommons.org/licenses/odbl/1.0/). The Open Database License (ODbL) is a license agreement intended to allow users to freely share, modify, and use this Dataset while maintaining this same freedom for others, provided that the original source and author(s) are credited.Author: Yongxiang Hou

Supplementary material 3 Table S3 Data type table Explanation note The best-fit partition schemes and model calculated by PartitionFinder v2.1.1. https://binary.pensoft.net/file/574785This dataset is made available under the Open Database License (http://opendatacommons.org/licenses/odbl/1.0/). The Open Database License (ODbL) is a license agreement intended to allow users to freely share, modify, and use this Dataset while maintaining this same freedom for others, provided that the original source and author(s) are credited.Author: Yongxiang Hou
