# Supplementary material for: Molecular and morphological evidence reveals a new genus of the subfamily Heteropterinae (Lepidoptera, Hesperiidae) from China
Source: Zookeys. 2021 Aug 5;1055:55–67. doi: 10.3897/zookeys.1055.68640 (PMC8360823; doi:10.3897/zookeys.1055.68640)
Supplement: Supplementary material 1 — Table S1 [file zookeys-1055-055-s001.docx]

**Table S1.** Information and GenBank accession number of the specimens used in this study.

| **Taxon** | **Locality** | **Voucher** | **Date** | **GenBank Accession Nos.** | | | |
| --- | --- | --- | --- | --- | --- | --- | --- |
|  |  |  |  | **COⅠ** | **EF-1α** | **RPS5** | **Wingless** |
| *Apostictopterus fuliginosus* | Xizang, China | SCAU He2001 | 2018-Ⅶ | MH985707^a^ | MZ165622 | MZ165653 | MZ165684 |
| *Antipodia atralba* | ‒ | 616MCZ^d^ | ‒ |  |  |  |  |
| *Argopteron aureipennis* | ‒ | BN000139^f^ | ‒ |  |  |  |  |
| *Argopteron puelmae* | Vilches Mto, Chile | SCAU He2263 | 2018-Ⅰ | MZ157817 | MZ165623 | MZ165654 | MZ165685 |
| *Astictopterus jama* | ‒ | 337ADW^d^ | ‒ |  |  |  |  |
| *Barca bicolor* | Xizang, China | SCAU He1664 | 2017-Ⅵ | MN199394^b^ | MZ165624 | MZ165655 | MZ165686 |
| *Burara aquilina* | ‒ | 383ADW^d^ | ‒ |  |  |  |  |
| *Butleria bissexguttatus* | ‒ | 629AVZB^d^ | ‒ |  |  |  |  |
| *Butleria elwesi* | ‒ | BN005146^f^ | ‒ |  |  |  |  |
| *Butleria flavomaculatus valdivianus* | Valdivia, Chile | NVG-16108G04^e^ | 1979-Ⅱ-24~25 |  |  |  |  |
| *Carterocephalus abax* | Yunnan, China | SCAU He2225 | 2019-Ⅷ-4 | MW115569^c^ | MZ165625 | MZ165656 | MZ165687 |
| *Carterocephalus alcina* | Yunnan, China | SCAU He2203 | 2016-Ⅶ-7 | MW115546^c^ | MZ165626 | MZ165657 | MZ165688 |
| *Carterocephalus argyrostigma* | Gansu, China | SCAU He2201 | 2020-Ⅵ-2 | MW115566^c^ | MZ165627 | MZ165658 | MZ165689 |
| *Carterocephalus avanti* | Xizang (Tibet), China | SCAU He1654 | 2018-Ⅴ-16 | MN199391^c^ | MZ165628 | MZ165659 | MZ165690 |
| *Carterocephalus dieckmanni* | Gansu, China | SCAU He2200 | 2020-Ⅵ-2 | MW115565^c^ | MZ165629 | MZ165660 | MZ165691 |
| *Carterocephalus houangty* | Sichuan, China | SCAU He2260 | 2019-Ⅵ-22 | MZ157818 | MZ165630 | MZ165661 | MZ165692 |
| *Carterocephalus longimaculatus* | Beijing, China | SCAU He2202 | 2014-Ⅴ-25 | MW115563^c^ | MZ165631 | MZ165662 | MZ165693 |
| *Carterocephalus palaemon* | Moscow, Russia | SCAU He2233 | 2017-Ⅲ-23 | MW115573^c^ | MZ165632 | MZ165663 | MZ165694 |
| *Carterocephalus patra* | Yunnan, China | SCAU He2221 | 2019-Ⅷ-4 | MW115568^c^ | MZ165633 | MZ165664 | MZ165695 |
| *Carterocephalus silvicola* | Kamikawa, Japan | SCAU He2230 | 2017-Ⅶ-20 | MW115574^c^ | MZ165634 | MZ165665 | MZ165696 |
| *Dalla agathocles* | Tungurahua, Ecuador | JU19001 | 2018-Ⅸ-27 | MZ157819 | MZ165635 | MZ165666 | MZ165697 |
| *Dalla cocha* | Peru | Dalla19 | 2020 | MZ157820 | MZ165636 | MZ165667 | MZ165698 |
| *Dalla epiphaneus* | Peru | Dalla7 | 2020 | MZ157822 | MZ165638 | MZ165669 | MZ165700 |
| *Dalla mesoxantha* | Naiayos, Peru | SCAU He2276 | 2017-Ⅲ | MZ157823 | MZ165639 | MZ165670 | MZ165701 |
| *Dalla vinca* | Peru | Dalla78 | 2020 | MZ157824 | MZ165640 | MZ165671 | MZ165702 |
| *Dalla wardi* | Peru | Dalla93 | 2020 | MZ157825 | MZ165641 | MZ165672 | MZ165703 |
| Dalla cypselus | Peru | Dalla2 | 2020 | MZ157821 | MZ165637 | MZ165668 | MZ165699 |
| *Dardarina aspila* | ‒ | BN004729^f^ | ‒ |  |  |  |  |
| *Dardarina dardaris* | Morelos, Mexico | JU19005 | 2018-Ⅶ-5 | MZ157826 | MZ165642 | MZ165673 | ‒ |
| *Dardarina dardaris* | ‒ | 198ADW^d^ | ‒ |  |  |  |  |
| *Erynnis afranius* | ‒ | 52ADW^d^ | ‒ |  |  |  |  |
| *Euschemon rafflesia* | ‒ | 85ADW^d^ | ‒ |  |  |  |  |
| *Freemaniana rawlinsi* | ‒ | BN003731^f^ | ‒ |  |  |  |  |
| *Heteropterus morpheus* | Jilin, China | SCAU He2240 | 2016-Ⅶ-15 | MZ157827 | MZ165643 | MZ165674 | MZ165704 |
| *Heteropterus morpheus* | ‒ | RE07G368^f^ | ‒ |  |  |  |  |
| *Hovala pardalina* | ‒ | BN003761^f^ | ‒ |  |  |  |  |
| *Hovala pardalina* | Fianarantsoa, Madagascar | NVG-7767^e^ | 1988-Ⅸ-13 |  |  |  |  |
| *Isoteinon lamprospilus* | ‒ | 165ADW^d^ | ‒ |  |  |  |  |
| *Ladda crithote* | Napo, Ecuador | JU19004 | 2018-Ⅸ-23 | MZ157828 | MZ165644 | MZ165675 | MZ165705 |
| *Ladda eburones eburones* | Cuzco, Peru | NVG-18014F02^f^ | 2008-ⅩⅠ-8 |  |  |  |  |
| Ladda ticidas ssp. | Zamora, Ecuador | JU19003 | 2017-Ⅸ-8 | MZ157829 | MZ165645 | MZ165676 | MZ165706 |
| *Lepella lepeletier* | ‒ | ME13E039^f^ | ‒ |  |  |  |  |
| *Leptalina unicolor* | Liaoning, China | SCAU He2239 | 2016-Ⅶ-9 | MZ157830 | MZ165646 | MZ165677 | MZ165707 |
| *Leptalina unicolor* | ‒ | BN000462^f^ | ‒ |  |  |  |  |
| *Metisella aegipan* | Verloren Valei, South Africa | SZSMETI013 | 2012-ⅩⅡ-8 | MZ157831 | MZ165647 | MZ165678 | MZ165708 |
| *Metisella kambove gamma* | Bamenda, Cameroon | SZSMETI003 | 2013-Ⅳ-13~16 | MZ157832 | MZ165648 | MZ165679 | MZ165709 |
| *Metisella metis paris* | The Downs, South Africa | SZSMETI011 | 2011-Ⅹ-14 | MZ157833 | MZ165649 | MZ165680 | MZ165710 |
| *Ochlodes sylvanoides* | ‒ | AW50^d^ | ‒ |  |  |  |  |
| *Piruna aea* | ‒ | 275ADW^d^ | ‒ |  |  |  |  |
| *Piruna pirus* | Colorado, USA | NVG-6454^e^ | 2016-Ⅶ-6 |  |  |  |  |
| *Pulchroptera pulchra* **comb. nov.** | Yunnan, China | SCAU He2223 | 2017-Ⅲ-23 | MZ157834 | MZ165650 | MZ165681 | MZ165711 |
| *Pulchroptera pulchra* **comb. nov.** | Yunnan, China | SCAU He2224 | 2017-Ⅲ-23 | MZ157835 | MZ165651 | MZ165682 | MZ165712 |
| *Tsitana tulbagha* | ‒ | ADW586^d^ | ‒ |  |  |  |  |
| *Urbanus dorantes* | ‒ | AW280^d^ | ‒ |  |  |  |  |
| *Willema willemi* | Loding, South Africa | SZSMETI008 | 2010-ⅩⅡ-11 | MZ157836 | MZ165652 | MZ165683 | ‒ |
| *Willema willemi* | ‒ | BN005142^f^ | ‒ |  |  |  |  |

^a, b, c, d, e, f^ indicate that gene sequences were downloaded from NCBI, and correspond to specimens in a: Han et al. 2018; b: Cao et al. 2019; c: Hou et al. 2021; d: Sahoo et al. 2016; e: Cong et al. 2019; f: Toussaint et al. 2020. ‒ indicates that the information was not available.
