# Supplementary material for: Molecular and morphological evidence reveals a new genus of the subfamily Heteropterinae (Lepidoptera, Hesperiidae) from China
Source: Zookeys. 2021 Aug 5;1055:55–67. doi: 10.3897/zookeys.1055.68640 (PMC8360823; doi:10.3897/zookeys.1055.68640)
Supplement: Supplementary material 2 — Table S2 [file zookeys-1055-055-s002.docx]

**Table S2.** Primers used in this study.

| **Name** | **Gene** | **F/R** | **Primer sequence(5’-3’)** | **Position*** | **Citation** |
| --- | --- | --- | --- | --- | --- |
| LCO1490 | COⅠ | F | GGTCAACAAATCATAAAGATATTGG | 1-658 (658bp) | Folmer *et al*., 1994 |
| HCO2198 |  | R | TAAACTTCAGGGTGACCAAAAAATCA |  | Folmer *et al*., 1994 |
| ef44 | EF-1α | F | GCYGARCGYGARCGTGGTATYAC | 1-1066 (1066bp) | Monteiro & Pierce, 2001 |
| efrcM4 |  | R | ACAGCVACKGTYTGYCTCATRTC |  | Monteiro & Pierce, 2001 |
| HybCho |  | F | GTCACCATCATYGACGC | 60-576 (517bp) | Wahlberg & Wheat, 2008 |
| HybVerdi |  | R | GACACCAGTTTCIACTCTGCC |  | Wahlberg & Wheat, 2008 |
| HybEF51.9 |  | F | CARGACGTATACAAAATCGG | 555-1066 (511bp) | Wahlberg & Wheat, 2008 |
| efrcM4 |  | R | ACAGCVACKGTYTGYCTCATRTC |  | Monteiro & Pierce, 2001 |
| RPS5F | RPS5 | F | ATGGCNGARGARAAYTGGAAYGA | 1-610 (610bp) | Wahlberg & Wheat, 2008 |
| RPS5R |  | R | CGGTTRGAYTTRGCAACACG |  | Wahlberg & Wheat, 2008 |
| 5R256F |  | F | ACTAACTCCCTGATGATGCA | 256-610 (355bp) | In this study |
| RpS5R |  | R | CGGTTRGAYTTRGCAACACG |  | Wahlberg & Wheat, 2008 |
| LepWG1 | wingless | F | GARTGYAARTGYCAYGGYATGTCTGG | 1-403 (403bp) | Warren *et al*., 2008 |
| LepWG2 |  | R | ACTICGCRCACCARTGGAATGTRCA |  | Warren *et al*., 2008 |
| Wg578F |  | F | TGCACNGTGAARACYTGCTGGATG | 29-403 (375bp) | Ward & Dwnie, 2005 |
| Wg1032R |  | R | ACYTCGCAGCACCARTGGAA |  | Abouheif & Wray, 2002 |
| Wg578F |  | F | TGCACNGTGAARACYTGCTGGATG | 29-340 (312bp) | Ward & Dwnie, 2005 |
| W340R |  | R | CGNCCGCARCACATRAGGTC |  | In this study |

*The position of the amplified fragments refers to the first set of primers for each gene.
