# Supplementary material for: Molecular and morphological evidence reveals a new genus of the subfamily Heteropterinae (Lepidoptera, Hesperiidae) from China
Source: Zookeys. 2021 Aug 5;1055:55–67. doi: 10.3897/zookeys.1055.68640 (PMC8360823; doi:10.3897/zookeys.1055.68640)
Supplement: Supplementary material 3 — Table S3 [file zookeys-1055-055-s003.docx]

**Table S3.** The best-fit partition schemes and model calculated by PartitionFinder v2.1.1.

| **Partition number** | **Optimal model** | **Partition strategy** | **Partition** |
| --- | --- | --- | --- |
| 1 | GTR+G | COI_pos1 | 1-658\3 |
| 2 | GTR+I+G | COI_pos2 | 2-658\3 |
| 3 | GTR+I+G | COI_pos3 | 3-658\3 |
| 4 | GTR+I+G | EF-1a_pos1 | 659-1724\3 |
| 5 | GTR+I+G | EF-1a_pos2 | 660-1724\3 |
| 6 | GTR+I+G | RPS5_pos3 EF-1a_pos3 | 1727-2334\3 661-1724\3 |
| 7 | GTR+I+G | RPS5_pos1 | 1725-2334\3 |
| 8 | GTR+I+G | RPS5_pos2 | 1726-2334\3 |
| 9 | GTR+G | wingless_pos1 | 2335-2737\3 |
| 10 | GTR+G | wingless_pos2 | 2336-2737\3 |
| 11 | GTR+G | wingless_pos3 | 2337-2737\3 |
